# Green fabrication of NiFe_2_O_4_@rGO nanocomposites for efficient electrochemical energy storage

**DOI:** 10.1039/d6ra05687c

**Published:** 2026-08-18

**Authors:** Naveed Aslam Dogar, Muhammad Pervaiz, Muhammad Shahbaz, Shahzad Sharif, Tayyaba Shahzadi, Irfan Ahmed Shaikh, Waheed Mushtaq, Tariq Mahmood

**Affiliations:** a Department of Chemistry, Government College University Lahore 54000 Pakistan mshahbazgcu@gmail.com; b Department of Chemistry, Government Graduate College of Science Wahdat Road Lahore Pakistan; c College of Earth and Environmental Science, University of the Punjab Lahore 54000 Pakistan

## Abstract

To meet the demands for sustainable and eco-friendly energy sources, researchers are focusing on the green synthesis of novel nanomaterials for energy storage. In the present study, nickel ferrite nanoparticles (NiF-NPs) were synthesized from the *Fagonia arabica* plant extract, followed by the formation of nickel ferrite nanocomposites with rGO (NiF-NCs). Structural characterization was performed by XRD, FTIR spectroscopy, SEM and EDX techniques. The electroanalytical tools, namely, CV, GCD and EIS, were employed to investigate the electrochemical properties of green-synthesized nanomaterials. NiF-NCs demonstrated improved electrochemical performance as compared to NiF-NPs. NiF-NCs exhibited a superior specific capacity (*Q*_s_) of 136.8 C g^−1^ at a current density of 1 A g^−1^. Dunn's graph showed diffusive and capacitive-controlled current contributions. To analyze the practical applications of NiF-NCs, they were used against an activated carbon (AC) electrode in an asymmetric supercapacitor device, where they delivered a remarkable *Q*_s_ of 100.6 C g^−1^ at 0.5 A g^−1^. The determined value of specific energy (*E*_s_) was 20.6 Wh kg^−1^, and the specific power (*P*_s_) was 835.9 W kg^−1^. A coulombic efficiency of 99% and a capacitance retention 40% were recorded after running 10 000 GCD cycles. The excellent electrochemical properties and cycling stability reported in this work highlight NiF-NCs as an attractive and efficient nanomaterial for future energy storage technologies.

## Abstract 2

Green fabrication of NiFe_2_O_4_@rGO nanocomposites for efficient electrochemical energy storage.

## Graphical abstract

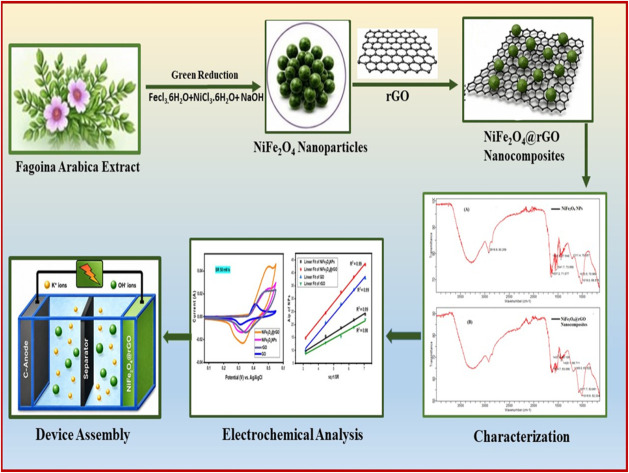

## 1. Introduction

Rapid industrialization and excessive consumption of fossil fuels to fulfill the energy requirements are raising concerns regarding environmental safety.^1,2^ The use of fossil fuels has led to climate change, air pollution, global warming and the loss of biodiversity. Researchers are focusing on sustainable and efficient ways to meet the rising energy needs by using renewable sources.^3,4^ Recent studies have focused more on the fabrication of an efficient material *via* a facile synthesis route and by exploring its practical applications.^5,6^ Hence, sodium-ion batteries, lithium-ion batteries and hybrid supercapacitors (HSCs)^7–9^ have emerged as the fields of significant interest. Earlier studies reported the synthesis of sustainable and cost-effective nanomaterials for energy storage.^10^ Batteries delivered higher specific energy (*E*_s_) and supercapacitors (SCs) offered higher specific power (*P*_s_). Thus, to overcome the limitations of *E*_s_ and *P*_s_, hybrid supercapacitors (HSCs) were fabricated.^11–13^ HSCs have gained major significance due to their high rate performance, low cost and remarkable cycling stability.^14^

Nanomaterials have exceptional physical and chemical properties with unique particle sizes, surface areas, shapes, compositions and dimensions that make them attractive materials for applications in drug delivery systems, biosensors, fuel cells, nano devices, supercapacitors and batteries.^15,16^ A variety of metal-based nanoparticles and nanocomposites were designed for energy storage applications because they offer more redox active sites, remarkable specific capacitance and high structural and cycling stability.^17^ Nanomaterials are synthesized by various methods like hydrothermal processes, chemical vapor deposition, mechanical milling and laser ablation, but these techniques are expensive with high energy consumption and limited control over the size of particles.^18,19^ Green synthesis by using plant extracts is an effective, sustainable, and inexpensive method for the preparation of nanoparticles.^20^ Different types of metal-based nanomaterials like spinel transition metal oxides have been reported. Among the spinel ferrites, nickel ferrite (NiFe_2_O_4_) nanoparticles are of significant interest. They have variable oxidation states, more than one coordination sites_,_ favorable surface-to-volume ratios and good chemical stability, which make them promising materials for energy storage applications.^21,22^ Several studies explored the electrochemical properties of NiF-NPs.^23^ NiF-NPs have moderate electrical conductivity, which can be improved by their integration with rGO, GO, CNT and many other carbon-derived materials. They offer better mechanical flexibility, high electrical conductivity, tunable ionic conductivity and excellent chemical stability. Hence, NPs are doped with various carbon-based functional materials to enhance the efficiency, capacity retention and cycling stability of electrodes for energy storage applications.^24,25^ GO is hydrophilic and efficient for surface functionalization, but due to its limited charge storage and slow charge transfer kinetics, GO is reduced to rGO. Because rGO has the tendency to enhance the electronic interaction and chemical stability of NPs. Phytoextracts were used as reducing agents to convert GO into rGO.^26^

To improve the stability and charge transfer activity of NPs, rGO was incorporated. NiFe_2_O_4_ NCs with rGO demonstrated a *C*_s_ of 584.63 F g^−1^ with 91% capacitance retention even after 5000 charge–discharge cycles.^27^ Singh *et al.* have explored the electrochemical response of NiFe_2_O_4_ NPs after their surface modification by using aryl diazonium salts. NPs after surface modification demonstrated a higher *C*_s_ of 1279 F g^−1^.^28^ NiFe_2_O_4_@S/C delivered an initial *Q*_s_ of 1193 mAh g^−1^ because of the incorporated rGO and CNTs.^29^ The incorporation of rGO increases the surface area, available active sites and remarkable electrochemical conductivity.^30^ Tahir *et al.* have reported the formation of N-rGO/NiFe_2_O_4_ with a smaller *R*_ct_ value and a *C*_s_ of 1727.81 F g^−1^.^31^ NiFe_2_O_4_@rGO offered an appreciable capacitance retention even after running 5000 cycles.^32^ rGO facilitated the electron transfer and offered a higher electrical conductivity than that of bare NPs.^1^ The studies have reported the improvement in electrochemical characteristics because of the synergistic effect of metal nanoparticles and rGO.^33^

The complex synthesis of NiFe_2_O_4_ nanoparticles has been reported in previous studies, which restricts the scalability. This research offers a novel approach to prepare NiF-NPs using an effective and environment-friendly green synthesis approach. By utilizing modified Hummer's method, GO was prepared. Then the reduction of GO into rGO was carried out by utilizing the extract of *Fagonia arabica*.^34^ Furthermore, the nanocomposites were prepared by the integration of rGO with NiF-NPs. The characterization was performed by employing XRD, FTIR spectroscopy, SEM and EDX. The electrochemical activity of NiF-NPs and NiF-NCs was explored by using CV, GCD and EIS for investigating their applications in energy storage. The novelty of the present work does not rely solely on combining nickel ferrite nanoparticles with rGO but also on integrating sustainable green synthesis with enhanced electrochemical performance. The main novelties of our work are as follows: green synthesis of NiFe_2_O_4_ nanoparticles using the *Fagonia arabica* extract without using any hazardous chemical reducing and stabilizing agents, eco-friendly reduction of graphene oxide using the plant extract rather than using toxic reducing agents, development of sustainable NiFe_2_O_4_@rGO nanocomposites *via* a plant-assisted synthesis route, fabrication of asymmetric supercapacitors (NiF-NCs//AC) with good cycling stability, high specific capacity and excellent coulombic efficiency (99%), and finally, comprehensive electrochemical investigation of both pristine nanoparticles and their nanocomposites with reduced graphene oxide through CV, GCD, EIS and Dunn's method.

## 2. Synthesis and method

### 2.1. Materials

Ferric chloride hexa-hydrate (FeCl_3_·6H_2_O), nickel chloride hexa-hydrate (NiCl_2_·6H_2_O), sodium hydroxide (NaOH), dried *Fagonia arabica* plant, distilled water, filter paper, *N*-methyl-2-pyrrolidone (NMP), hydrochloric acid (HCl), activated carbon (AC), polyvinylidene fluoride (PVDF), aniline and potassium hydroxide (KOH). All the chemicals and the solvents were procured from Sigma-Aldrich and used without further treatment or purification.

### 2.2. Plant extraction

For preparing plant extract, 25 grams of *Fagonia arabica* powder was mixed in 500 mL of distilled water and then stirred at 4500 rpm for 5 h on a hot plate. During this process, the temperature was kept at 60 °C, and the solution was separated through filtration. Finally, the filtrate was stored for further use.^35^*Fagonia arabica* extract contains naturally occurring phytochemicals like flavonoids, alkaloids, phenolic compounds, tannins, terpenoids, reducing sugars and glycosides. All these phytochemicals contain hydroxyl and carboxyl functional groups, which play a vital role in reduction and stabilization during nanoparticle synthesis.

### 2.3 Green synthesis of nickel ferrite nanoparticles (NiF-NPs)

First, 100 mL of 0.2 M NiCl_2_·6H_2_O solution was mixed in 100 mL of 0.4 M FeCl_3_·6H_2_O solution. The solutions after mixing were stirred on a hot plate at room temperature. Then, 25 mL of prepared solution of the *Fagonia arabica* green extract was added dropwise into the above solution. The whole mixture was mixed for about 30 minutes through constant stirring. The synthesis of NiF-NPs was confirmed by color change. After the solution was allowed to stand overnight, a slurry was observed at the bottom. However, washing was performed several times with water and, finally, with ethanol. After that, the nanoparticles were dried at 70 °C in an oven. The nanoparticles were collected in vials in a powdered form. It is important to mention that the phytochemicals in *Fagonia arabica* plants contain reducing, capping and stabilizing agents. Phenolic and hydroxyl groups help in reducing metal ions into nano-sized particles, while tannins, flavonoids and other biomolecules adsorb onto the surface and prevent excessive agglomeration and control particle growth. All these steps eliminate the use of hazardous reducing agents and make the entire process environmentally benign.

### 2.4 Preparation of graphene and reduced graphene oxide

Modified Hummer's method was employed to prepare graphene oxide from graphite flakes. In the method, graphite was oxidized in concentrated H_2_SO_4_ by using KMnO_4_ as an oxidizing agent under well-controlled conditions. H_2_O_2_ was employed to terminate the reaction and GO was obtained, which was washed with distilled water and 10% HCl to remove residual impurities before drying at 80 °C for 8 h.^36^ However, reduced graphene oxide (rGO) was prepared by reducing graphene oxide using the plant extract. For this purpose, the GO-plant extract mixture was stirred at 70 °C for 1 h, followed by centrifugation, washing with water and ethanol and finally drying for 8 h at 80 °C. The final product obtained was reduced graphene oxide.

### 2.5 Green synthesis of nickel ferrite-reduced graphene oxide nanocomposites (NiF-NCs)

**Fig. 1:**
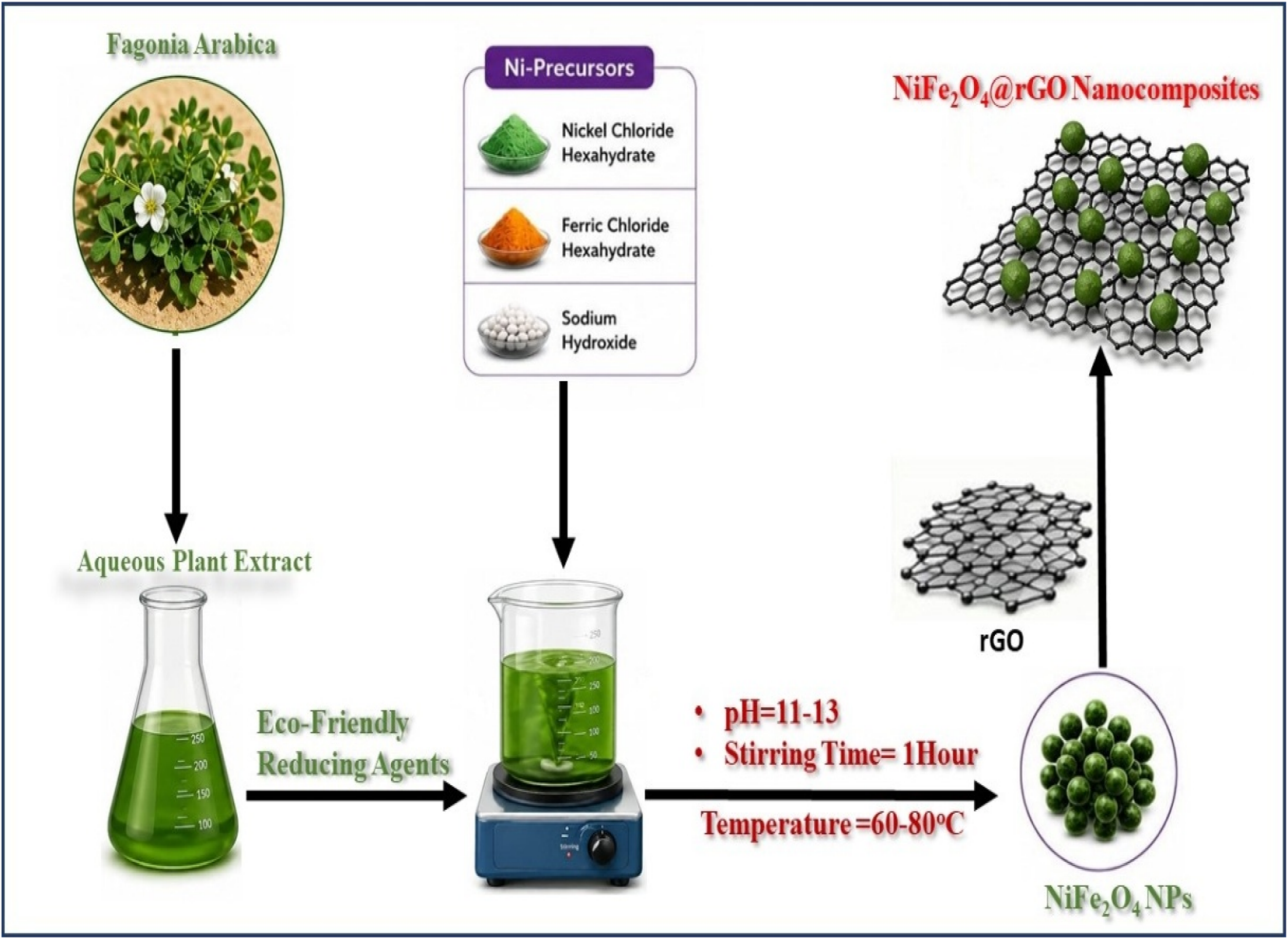
Schematic of the synthesis of NiF-NPs and NiF-NCs.

First, 50 mg of reduced graphene oxide and 50 mg of nickel ferrite nanoparticles were taken in separate beakers and sonicated in an ultrasonicator. The reduced graphene oxide solution was stirred continuously on a hot plate for 3 h while adding the solution of nickel ferrite nanoparticles into it. After that, a homogeneous suspension was prepared and poured into a Teflon-lined autoclave. The autoclave was kept in an oven at 150 °C for 24 h. After this period, the autoclave was taken out from the oven and cooled to room temperature. The dried NiFe_2_O_4_@rGO nanocomposites were calcined at 400 °C for 4 hours at a rate of 5 °C per minute (Fig. 1).

### 2.6. Electrode fabrication and electrochemical measurement

To investigate the electrochemical properties of NiF-NPs and NiF-NCs, CV, GCD and EIS analyses were performed. In a three-electrode assembly, Ag/AgCl served as the reference electrode, a Pt wire served as the auxiliary/counter electrode and the third was the working electrode. For the fabrication of the working electrode, a slurry was formulated by thoroughly mixing NiF-NPs (80%), PVDF (10%) and AC (10%) in an NMP solvent. The slurry was kept under stirring for 6–8 h to obtain a uniform composition. A nickel foam (NF) of 2 mm thickness was cut (∼1 × 1.5 cm^2^) for slurry deposition. The NF was rinsed with hydrochloric acid (HCl), distilled H_2_O and ethanol (C_2_H_5_OH), respectively. The slurry was deposited on the NF by drop-casting. The electrode was placed in an oven for 4 h at 70 °C for drying. The electrode was named NiFe_2_O_4_@AC (NiF-NPs). The same method was used to prepare the electrode of NiF-NCs using rGO instead of AC, and the electrode was named NiFe_2_O_4_@rGO (NiF-NCs). The mass loading was 4 mg on each electrode.

In three-electrode assembly, CV was conducted within a specific potential window (0–0.55 V) at discrete scan rates (10–50 mV s^−1^). GCD was performed at different current densities (1–5 A g^−1^) to analyze the rate capability and charge storage behavior of the electrode. The *Q*_s_ values determined in three-electrode and two-electrode assemblies were based solely on the active material's mass loading. To test the electrode stability, 10 000 GCD cycles were run. The EIS technique was performed within a specific frequency range (100 mHz–100 kHz). The electrochemical characteristics of the NiF-NCs electrode were tested in a hybrid supercapacitor assembly, using a 1 M KOH solution as the electrolyte. For the optimization of the asymmetric device performance, mass and charge were balanced using eqn (1):^37^1
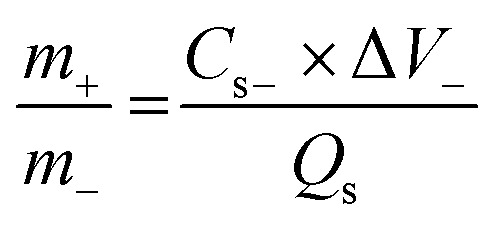
where *Q*_s_ denotes the specific capacity and ‘*m*’ the active material's loaded mass on positive and negative electrodes. Δ*V* corresponds to the operating potential window (PW) and *C*_s_ represents the specific capacitance of the negative electrode. By employing eqn (2) and (3), the specific capacity (C g^−1^) and specific capacitance (F g^−1^) were obtained. The energy density (Wh kg^−1^) was calculated by applying eqn (4) and the power density (W kg^−1^) was determined by using eqn (5):2
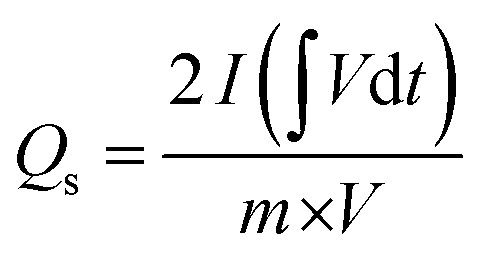
3
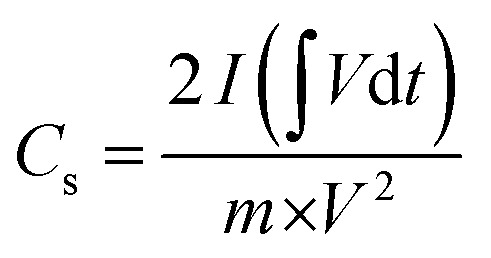
4
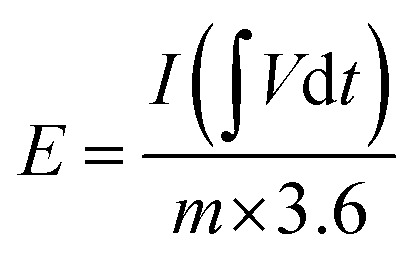
5
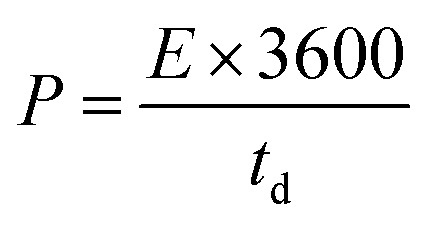
where *t*_d_ corresponds to the discharge time and *I*/*m* refers to the current density.

The underlying process of charge storage was elucidated by calculating the *b*-value using the power law (eqn (6) and (7)):

6 *i* = *av*^*b*^

7 log(*i*) = log(*a*) + *b* log(*v*)

where ‘*v*’ corresponds to the scan rate, ‘*a*’ is a constant, which can be adjusted, ‘*i*’ signifies the peak current and ‘*b*’ represents the slope. The electrochemical work station of OrigaLys by utilizing OrigaFlex-OFF05A and OrigaFlex-OGFEIS was used for electrochemical analysis. Built-in Randles circuit fitting was utilizing to calculate the EIS attributes.

## 3. Results and discussion

### 3.1. Structural characterization

#### 3.1.1. X-ray diffraction analysis

**Fig. 2:**
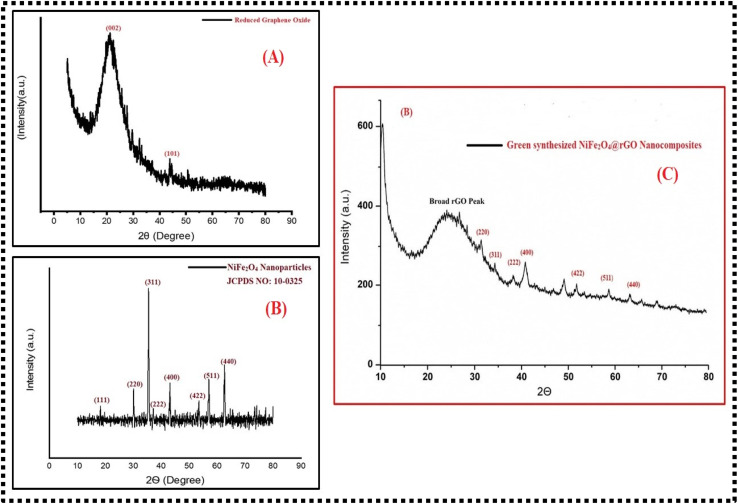
XRD spectra of (A) rGO, (B) NiF-NPs and (C) NiF-NCs.

The XRD pattern of reduced graphene oxide (rGO) is shown in Fig. 2A. The broad diffraction peak at approximately 24–26° (2*θ*) corresponds to the (002) plane of graphitic carbon. The presence of this broad peak suggests the partial restoration of graphitic structure following the reduction of graphene oxide. The absence of graphene oxide characteristic peak around 10°–12° (2*θ*) further confirms the successful removal of a significant portion of oxygen-containing functional groups due to reduction. The broad nature of the (002) reflection suggests a low degree of crystallinity, incomplete restacking of graphene layers, and the presence of structural defects, which are characteristic features of reduced graphene oxide. Besides this, weak diffraction features are observed in the 40°–50° (2*θ*) region, which can be attributed to the (101) plane of graphitic carbon, indicating the partial restoration of the sp^2^-hybridized carbon network. The relatively low intensity and broadness of these reflections further support the disordered structure of rGO. The XRD results suggest the successful reduction of graphene oxide and the formation of reduced graphene oxide with a partially restored graphitic framework and a defect-rich layered morphology.

The XRD patterns of NiF-NPs and NiF-NCs in Fig. 2B and C confirm the successful green synthesis of nanocrystalline nickel ferrite with inverse spinal cubic structure and their integration with reduced graphene oxide. The diffraction peaks of nanoparticles at (111), (220), (311), (222), (400), (422), (511) and (440) planes match well with the standard JCPDS card No. 10-0325 corresponding characteristic planes of cubic nickel ferrite nanoparticles.^38^ The broadening of diffraction peaks indicates the presence of small crystallites and partial amorphous nature arising due to plant-assisted synthesis because biogenic agents reduce the crystal growth. In the nanocomposite spectrum (Fig. 2C), a broad hump appearing around 20°–30° is attributed to reduced graphene oxide. The increase in broadness and the suppression of ferrite reflections after rGO incorporation suggests strong interaction between NiFe_2_O_4_ nanoparticles and reduced graphene oxide, thus confirming the successful synthesis of NiFe_2_O_4_@rGO nanocomposites. This successful integration helps in reducing the agglomeration and increasing the dispersion of nanoparticles on the reduced graphene oxide matrix. These structural modifications are expected to improve the electron transport behaviour, thus making the nanocomposites promising candidates for electrochemical and photocatalytic applications.^39^ According to the Debye–Scherrer equation, the sizes of pristine nanoparticles and rGO-integrated nanocomposites were 61 and 72 nm, respectively. The Debye–Scherrer equation which was used is as follows:

8 *D* = *Kλ*/*β* Cos *θ*

where *D* is the average crystallite size (nm), *K* represents the shape factor, *λ* denotes the wavelength of the X-ray source, *β* is the full width at half maximum (FWHM) and *θ* is Bragg's angle.

#### 3.1.2. FTIR analysis

**Fig. 3:**
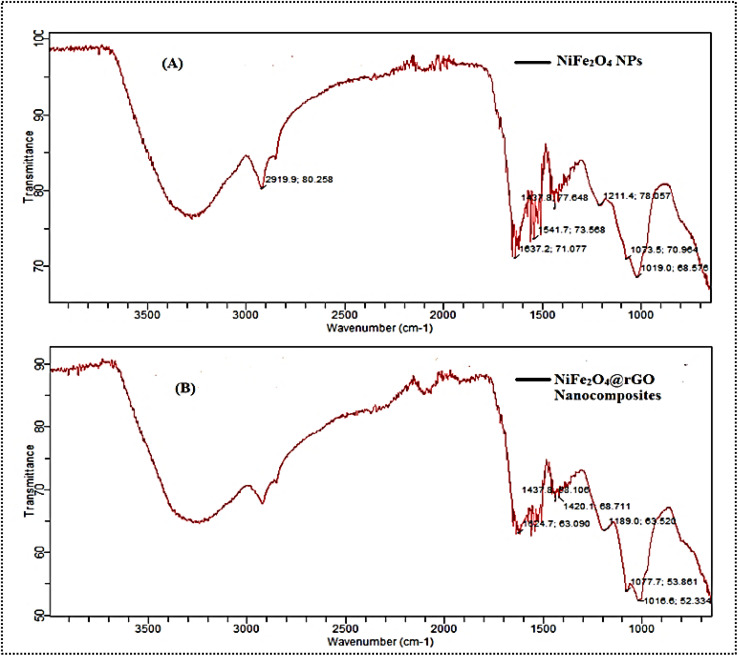
FTIR spectrum of green-synthesized (A) NiF-NPs and (B) NiF-NCs.

The FTIR spectrum of NiF-NPs (Fig. 3A) show a broad absorption band in the 3200–3500 cm^−1^ region, which is assigned to the O–H stretching vibrations of surface hydroxyl groups. The weak band at 2919 cm^−1^ corresponds to the C–H stretching, and is related to minor organic residues due to plant-assisted synthesis. The absorption bands around 1600–1400 cm^−1^ are attributed to the C=C- and C–O-related vibrations and surface-bound phytochemical groups. The peaks around 1211, 1073 and 1019 cm^−1^ are ascribed to the C–O and C–O–C functional groups. However, the peaks below 700 cm^−1^ are most important and are assigned to the Ni–O and Fe–O stretching vibrations, confirming the formation of spinel nickel ferrite.^38^ The FTIR spectrum of NiF-NCs shows a broad O–H band but with slight modifications, which suggests the interaction between the nanoparticles and the rGO surface (Fig. 3B). The bands near 1437, 1368, 1189, 1079 and 1018 cm^−1^ indicate the presence of C=C graphitic vibrations and the residual C–O/C–O–C groups of rGO and organic residues.^40^ The retention of Ni–O vibrations confirms that the spinel ferrite structure remains intact after nanocomposite formation. In the FTIR spectrum of nanocomposites, slight shifting and intensity reduction of the peaks as compared to the pristine nanoparticles confirm the successful interaction and anchoring of the nanoparticles on rGO sheets.

#### 3.1.3. SEM and EDX analysis

**Fig. 4:**
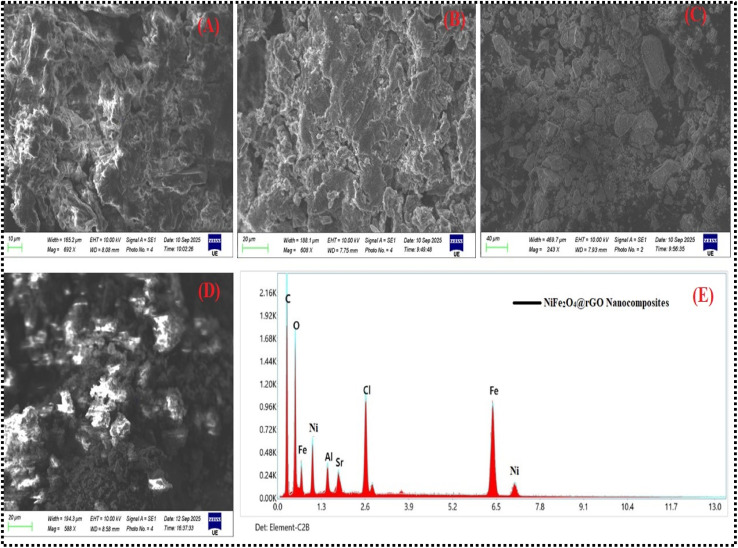
(A) SEM image of graphene oxide. (B) SEM image of reduced graphene oxide (rGO). (C) SEM image of green-synthesized nickel ferrite nanoparticles (NiFe_2_O_4_ NPs). (D) SEM of green-synthesized nickel ferrite-reduced graphene oxide (NiFe_2_O_4_@rGO) nanocomposites. (E) EDX spectrum of green-synthesized nickel ferrite-reduced graphene oxide (NiFe_2_O_4_@rGO) nanocomposites.

SEM and EDX analyses were performed to investigate the surface morphology and elemental composition of the synthesized materials, respectively. The SEM image of graphene oxide (GO) indicates a layered and wrinkled sheet-like morphology and the presence of stacked flakes, thus confirming the successful oxidation of graphite layers (Fig. 4A). While the crumpled, porous and corrugated structure of reduced graphene oxide (rGO) reveals the removal of oxygen-containing functional groups due to successful reduction (Fig. 4B). This reduction also plays a vital role in enhancing the surface area and roughness.^41^ Nearly spherical and agglomerated nanostructures of NiF-NPs can be seen (Fig. 4C). This agglomeration is possibly due to the magnetic interaction and high surface energy of ferrite nanoparticles.^38^ Furthermore, successful anchoring and nearly uniform distribution of NiF-NPs on reduced graphene sheets can be observed, thus forming an interconnected conductive network (Fig. 4D). The incorporation of reduced graphene oxide effectively reduces the nanoparticle agglomeration and provides a high-surface-area matrix for nanoparticle dispersion, which makes these nanocomposites effective for electrochemical and photocatalytic applications.^39^ Besides, the EDX spectrum of NiFe_2_O_4_@rGO nanocomposites further verifies the elemental composition of the green-synthesized nanocomposites by showing peaks of Ni, Fe, O and C elements (Fig. 4E). Strong carbon peaks confirm the successful integration between NiFe_2_O_4_ nanoparticles and rGO. The absence of significant impurity peaks indicates the pure nature of the synthesized nanomaterials. Minor traces of Cl, Al, and Sr indicate plant extract impurities and instrumental articrafts. Overall, SEM and EDX analyses collectively confirm the successful synthesis of NiFe_2_O_4_ nanoparticles and their effective integration with reduced graphene oxide to synthesize NiF_2_O_4_@rGO nanocomposites.

### 3.2. Electrochemical characterization

Electrochemical characterization was performed on the electrochemical work station of OrigaLys by utilizing OrigaFlex-OFF05A and OrigaFlex-OGFEIS.

#### 3.2.1. Half-cell electrochemical characterization

##### 3.2.1.1. Cyclic voltammetry (CV)

CV is an efficient and simple analytical tool employed to gain insights into the kinetics of oxidation–reduction processes, the electrochemical properties of the material and the thermodynamics of redox reactions. In the three-electrode assembly, electrochemical evaluation of NiF-NPs and NiF-NCs was carried out using 1 M KOH. The selection of a suitable electrolyte is a crucial factor because it significantly affects the device performance. An optimized concentration of electrolyte facilitates the movement of ions, reduces the resistance offered by the solution and improves the efficiency of the device. Although the increased concentration of the electrolyte enhances the current response and conductivity of ions, it results in a more viscous solution. A concentrated KOH solution masks the redox processes and hinders the ion diffusion into the electrode.^14^ An electrolyte solution of 1 M KOH is preferably used because it gives optimized results.

**Fig. 5:**
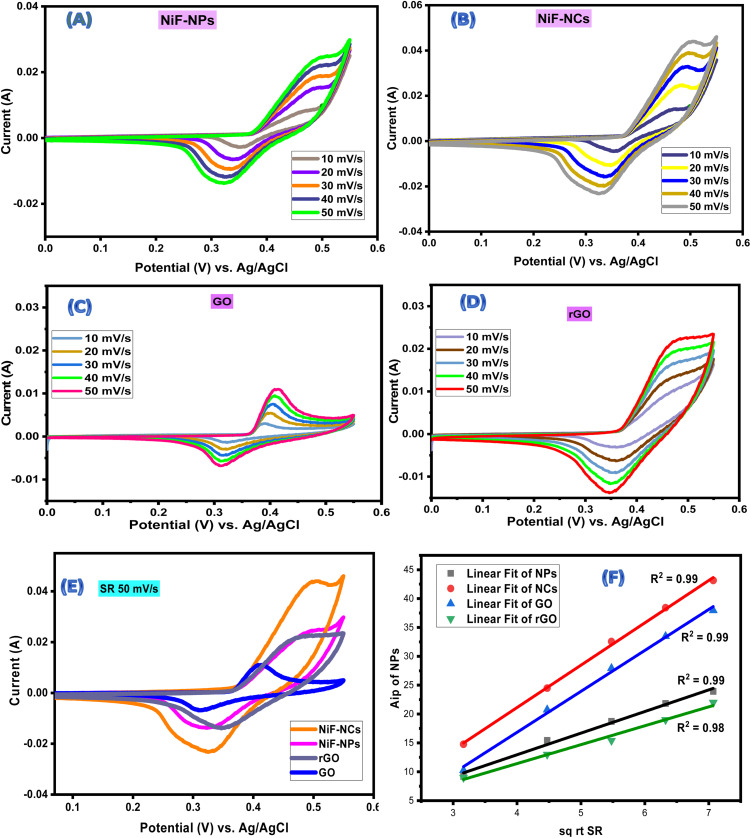
Cyclic voltammogram of (A) NiF-NPs, (B) NiF-NCs, (C) GO and (D) rGO at different scan rates. (E) Comparison of the CV plots of NiF-NPs, NiF-NCs, GO and rGO at 50 mV s^−1^ (F). Plot of the square root of the scan rate *versus* anodic peak currents displaying the values of regression coefficient (*R*^2^) for NiF-NPs, NiF-NCs, GO and rGO.

CV curves were recorded at several scan rates (10 to 50 mV s^−1^) in a PW range of 0 V to 0.55 V. The CV curves of NiF-NPs, NiF-NCs, GO and rGO are illustrated in Fig. 5A–D. The redox peaks in CV were allocated to the reversible redox transitions of Ni^2+/^Ni^3+^ and Fe^2+^/Fe^3+^ redox couples. As the scan rates were increased, a rise in peak currents was recorded. The peak separation reflects the reversible process and appreciable charge transfer kinetics. A comparison of the cyclic voltammograms of NiF-NPs, NiF-NCs, GO and rGO at 50 mV s^−1^ is displayed in Fig. 5E. The electrochemical performance in CV increased in the order of GO < rGO < NiF-NPs < NiF-NCs. Fig. 5F shows the plot of regression coefficient (*R*^2^). The determined value of *R*^2^ is 0.99, which indicates excellent reversibility.

**Fig. 6:**
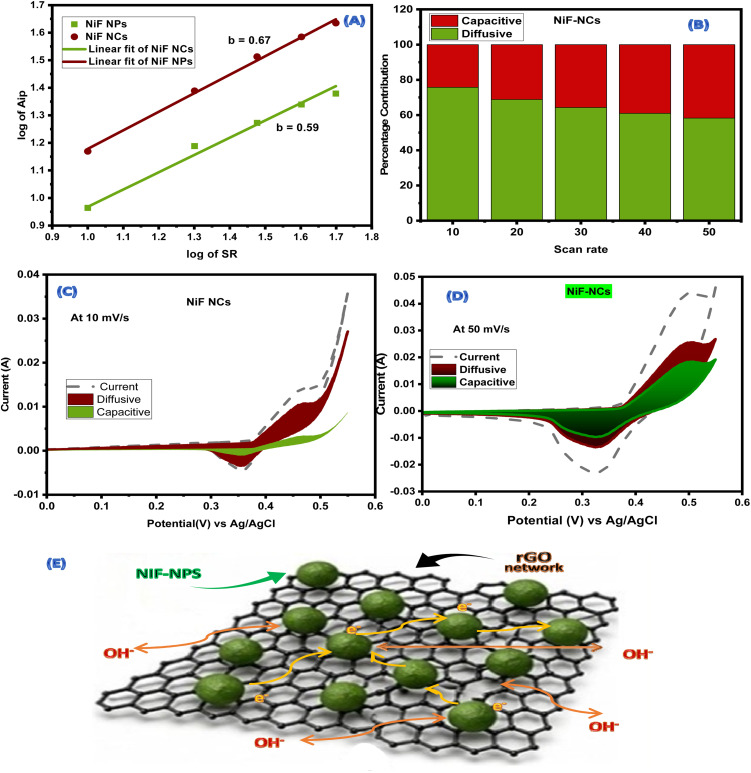
(A) Plot of the comparison of *b*-values for NiF-NPs and NiF-NCs. (B) Bar graph of NiF-NCs representing the variation in the percentage contribution of diffusive/capacitive currents at different scan rates. (C) Illustration of diffusive, capacitive and experimental CV responses at 10 mV s^−1^ (D) and 50 mV s^−1^. (E) Mechanistic insight into charge storage.

The increase in the scan rate will shift anodic peak current (*A*_ip_) towards a higher potential because of the internal resistance and limited time for ions diffusion.^42,43^ NiF-NCs showed higher peak current, larger area under the CV curve and smaller peak separation (Δ*E*_p_), indicating the enhanced specific capacity, fast charge transfer kinetics and smaller resistance.^44^ NiF-NCs showed improved results due to doping of rGO, because rGO has high surface area, more available active sites and good electrical conductivity. Thus, NiF-NCs showed better redox activity because of the synergistic effect of NiF-NPs with incorporated rGO. A plot of log of scan rate on the *x*-axis and log of *A*_ip_ on the *y*-axis was constructed to find the *b*-value. The *b*-value assists us to differentiate between the battery and capacitive-like charge storage processes. The determined *b*-value for NiF-NPs was 0.59 and that for NiF-NCs was 0.67, as presented in Fig. 6A. The cyclic voltammogram validated the predominant redox-type behavior of both electrodes. Additionally, Dunn's graphs were plotted to represent the diffusive- and capacitive-regulated currents. As the scan rates were increased from 10 mV s^−1^ to 50 mV s^−1^, the diffusive contribution decreased from 75% to 58%, and the capacitive-controlled current contributions increased from 24% to 41% (Fig. 6B). The capacitive and faradaic responses of the electrode along with the experimental value at 10 mV s^−1^ and 50 mV s^−1^ are illustrated in Fig. 6C and D, respectively. The mechanistic insight into charge storage is shown in Fig. 6E, which represents the faradaic pseudocapacitive behavior of NiF-NCs, enhanced by the incorporation of rGO.

##### 3.2.1.2. Galvanostatic charge–discharge (GCD)

**Fig. 7:**
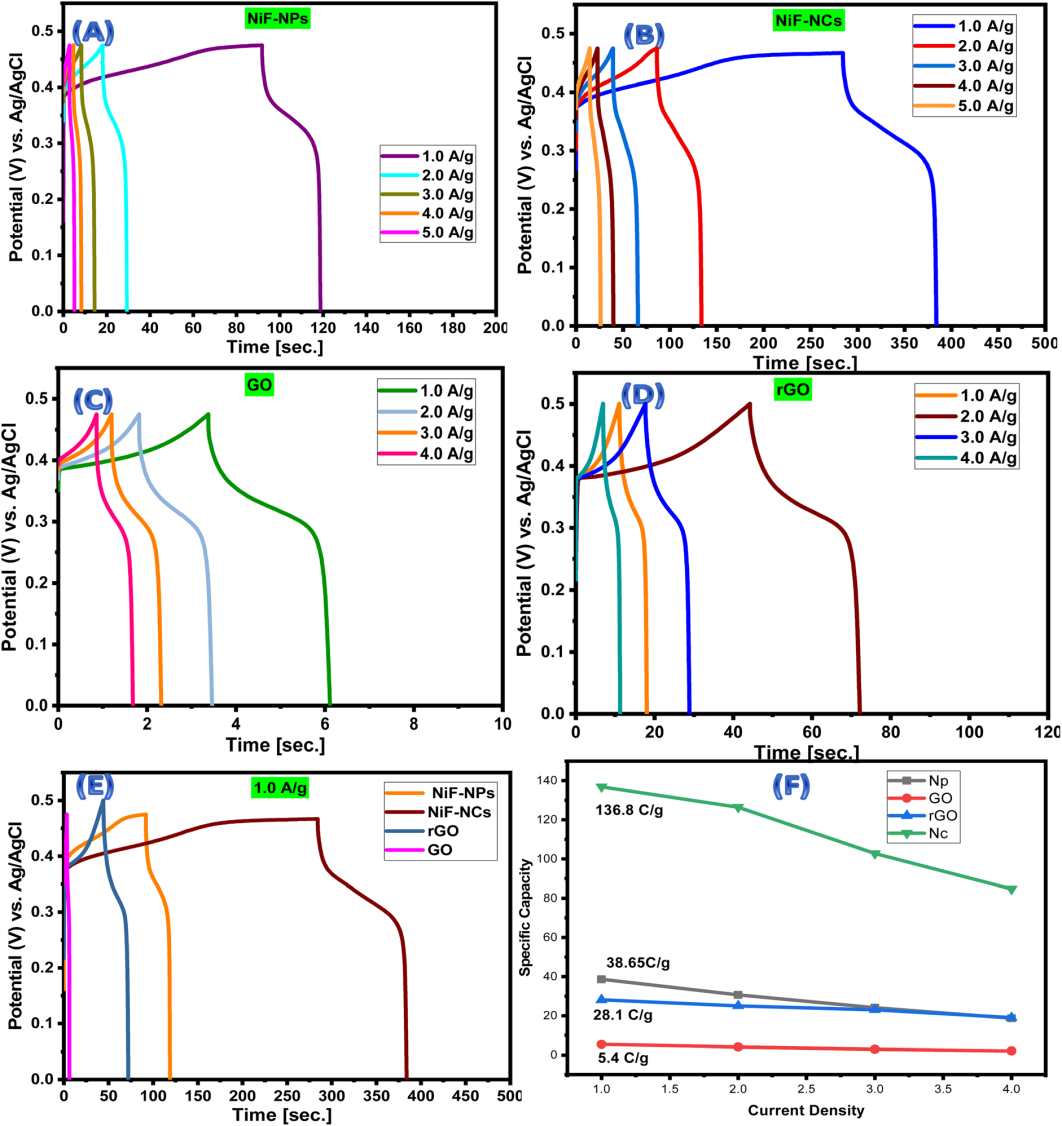
Galvanostatic charge–discharge plateaus of (A) NiF-NPs, (B) NiF-NCs, (C) GO and (D) rGO at different current densities. (E) Comparison of the GCD response of NiF-NPs, NiF-NCs, GO and rGO at 1.0 A g^−1^. (F) Comparison of the *Q*_s_ of NiF-NPs, NiF-NCs, GO and rGO at different current densities.

Galvanostatic charge–discharge was performed, from which the stability, *Q*_s_, *C*_s_ and efficiency of the device can be determined. The GCD data were acquired at a current density of 1 A g^−1^ to 5 A g^−1^ to test the electrode's energy behavior and stability. The GCD curves of NiF-NPs and NiF-NCs are presented in Fig. 7A and B, respectively. During the charging phase, a continuous rise in voltage was observed, displaying the GCD plateaus, which remained on the reversal of the applied current. The GCD curves of GO and rGO are illustrated in Fig. 7C and D. The impedance values were quantified by utilizing the built-in analysis software OrigaFlex-OGFEIS and OrigaFlex-OFF05A on an OrigaLys potentiostat. A comparison of the GCD analysis of NiF-NPs, NiF-NCs, GO and rGO at 1 A g^−1^ is shown in Fig. 7E. The distinct non-linear regions in GCD analysis indicated the battery-type behavior of the electrode materials. NiF-NPs and NiF-NCs demonstrated *Q*_s_ values of 38.65 C g^−1^ and 136.8 C g^−1^ at 1 A g^−1^, respectively (Fig. 7F). The *Q*_s_ values exhibited by GO and rGO were 5.4 C g^−1^ and 28.1 C g^−1^, respectively, at a current density of 1 A g^−1^, as shown in Fig. 7F. Moreover, the specific capacitance (*C*_s_) was determined as 81.38 F g^−1^ and 288.17 F g^−1^ for NiF-NPs and NiF-NCs at 1 A g^−1^, respectively. At a higher current density, the *Q*_s_ value decreased because the time period was limited for the interaction between the ions and the electrode. NiF-NCs showed the longest *t*_d_ at 1 A g^−1^ and the highest *Q*_s_ that reflects the superior performance of NiF-NCs for applications in energy storage devices.

##### 3.2.1.3. Electrochemical impedance spectroscopy (EIS)

**Fig. 8:**
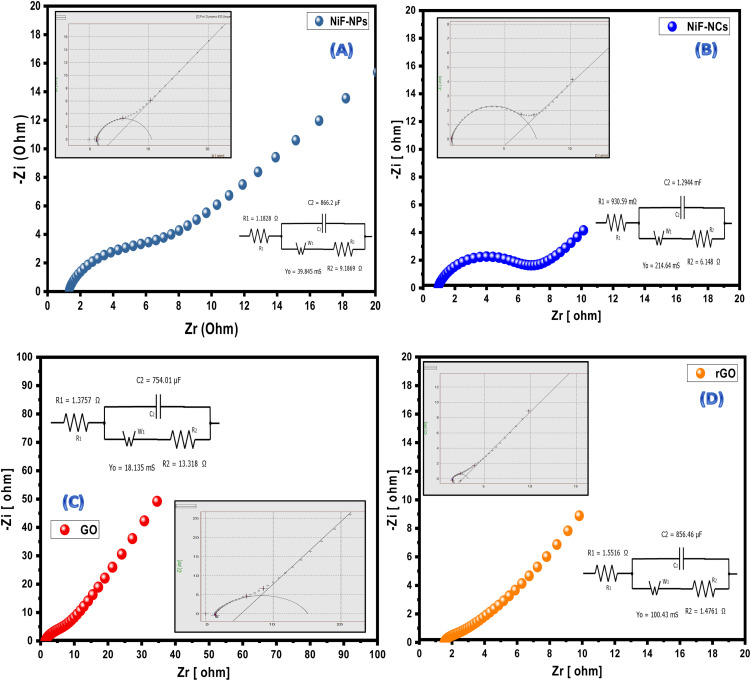
EIS spectrum and Randles circuits of (A) NiF-NPs, (B) NiF-NCs, (C) GO and (D) rGO in the three-electrode assembly with circuit fitting in insets.

To assess the electrical conductivity of NiF-NPs and NiF-NCs, EIS was performed. The EIS spectra provide insights into the ongoing electrochemical processes, such as interfacial resistance, mass transfer of redox active material towards the electrode surface and kinetics of charge transfer. EIS was performed in an alternating frequency varying from 100 mHz to 100 kHz. The Nyquist plots and Randles circuits of NiF-NPs, NiF-NCs, GO and rGO were presented in Fig. 8A–D. On the *x*-axis, the ESR value was recorded at the point of intersection. The ESR value for NiF-NPs was 1.18 Ω and that for NiF-NCs was 930.59 mΩ. The low ESR value of NCs reflects the enhanced conductivity and effective movements of ions through the electrolyte. The *R*_ct_ values in a semicircular high-frequency region were 9.18 Ω and 6.14 Ω for NiF-NPs and NiF NCs, respectively. The smaller *R*_ct_ value of NiF-NCs showed the better charge transfer kinetics of the electrode. Warburg impedance was represented in the low-frequency domain by a line at an angle of 45°, showed a value of 39.84 mS for NiF-NPs and 214.64 mS for NiF-NCs. The low resistance and better conductivity indicated the improved and appreciable storage behavior of NiF-NCs.

#### 3.2.2. Battery-supercapacitor hybrid assembly

**Fig. 9:**
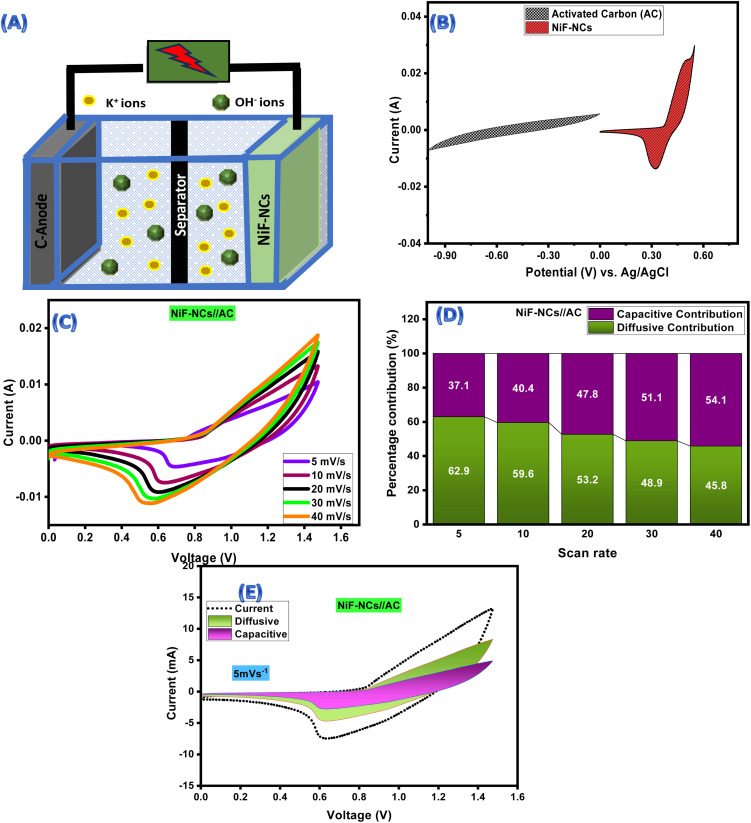
Electrochemical characterization of NiF-NCs in an HSC device: (A) Illustration of the NiF-NCs//AC hybrid assembly. (B) CV plots of AC along with NiF-NCs using a three-electrode (3E) setup. (C) CV profiles of NiF-NCs//AC at multiple scan rates. (D) Bar chart demonstrating the capacitive and diffusive current contributions (%) for NiF-NCs//AC at different scan rates. (E) Current contributions by capacitive- and diffusive-regulated processes for NiF-NCs//AC at 5 mV s^−1^.

To analyze the application potential of NiF-NCs, an HSC assembly was constructed, in which the working electrode behaved as the positive electrode and was run against the activated carbon electrode, which served as the negative electrode. An optimized concentration of KOH electrolyte (1 M) was used. The hybrid device (NiF-NCs//AC) is presented in Fig. 9A. In the three-electrode assembly, the AC shows rectangular CV curves in an optimized operating PW varying from −1 V to 0 V (Fig. 9B). The CV was performed at different scan rates (5–40 mV s^−1^) within a potential sweep in the range of 0 V to 1.475 V (Fig. 9C). The cyclic voltammogram of NCs demonstrated the pseudocapacitive nature of the device. The maintenance of CV curves at 40 mV s^−1^ depicted the good rate capability of NiF-NCs. The increase in scan rates results in increased capacitive contributions (in green color) and decreased diffusive contributions (in purple color), leading to the predominant EDLC behavior, as displayed in Fig. 9D. Dunn's method was employed to quantify the capacitive and diffusive current contributions. The graphical representation of the diffusive- and capacitive-regulated current contributions is displayed, where the dotted line corresponds to an experimental value at 5 mV s^−1^ (Fig. 9E). Thus, CV analysis validated the hybrid charge storage mechanisms and good reversibility of the device.

**Fig. 10:**
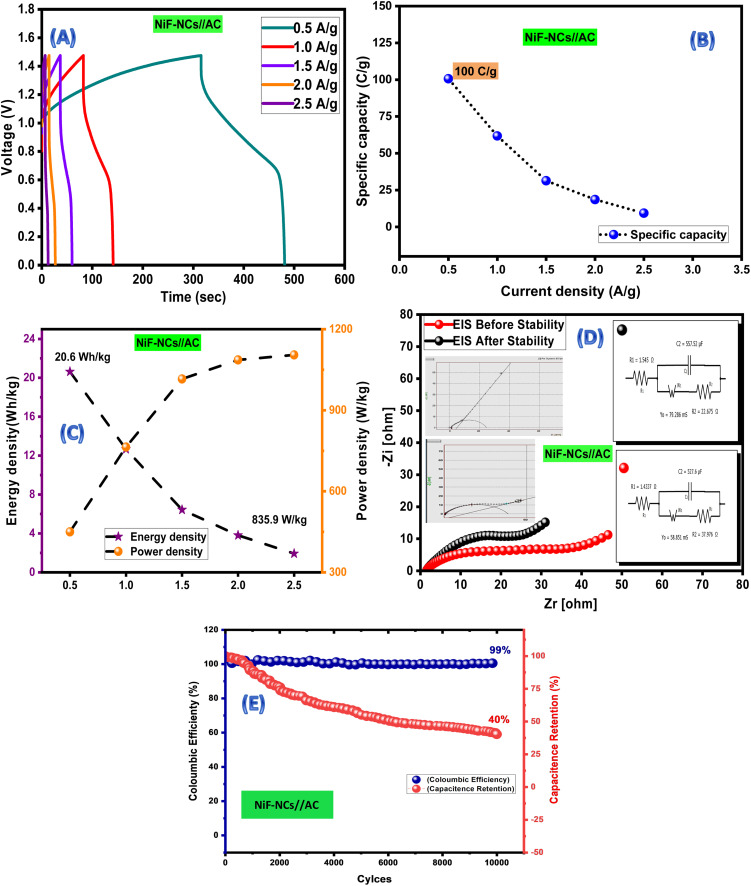
NiF-NCs HSC assembly: (A) GCD analysis of NiF-NCs//AC at different current densities. (B) Plot of the specific capacity (C g^−1^) of NiF-NCs//AC as a function of current densities. (C) Plot displaying the specific power and specific energy of the device at multiple current densities. (D) Illustration of the EIS results and circuit fitting insets of NiF-NCs//AC before and after the stability test. (E) Plot depicting the coulombic efficiency and capacitance retention of the NiF-NCs//AC device for 10 000 charge–discharge cycles.

GCD was carried out to evaluate the practical applications of the device at a current density in the range of 0.5–2.5 A g^−1^, as displayed in Fig. 10A. Asymmetrical GCD curves demonstrated that NiF-NCs exhibited both faradaic and non-faradaic reactions, which reflected the contributions of both redox and surface-controlled processes. The *Q*_s_ determined for NCs was 100 C g^−1^ at 0.5 A g^−1^ (Fig. 10B). The efficiency and performance of device were investigated by the *E*_s_ and *P*_s_. The device offered a maximum *E*_s_ of 20.6 Wh kg^−1^ at 0.5 A g^−1^ and an appreciable *P*_s_ of 835.9 W kg^−1^ at 2.5 A g^−1^ (Fig. 10C).

EIS analysis was utilized to investigate the energy storage behavior and electrical conductivity of the electrode. The Nyquist plots along with Randles circuits before and after performing the stability test are presented in Fig. 10D. *R*_ct_ was recorded as 37.97 Ω, which decreased to 22.67 after stability. The device showed a CE of 99% and a capacitance retention of 40% after 10 000 charge–discharge cycles (Fig. 10E).

## 4. Conclusions

This research work illustrated the successful synthesis of NiF-NPs and their composites with rGO by an eco-friendly and sustainable green synthesis method. The synthesized nanoparticles were characterized by XRD, FTIR spectroscopy, SEM and EDX techniques. Subsequently, CV, GCD and EIS analyses were performed to explore the electrochemical properties of NPs and NCs. In a three-electrode assembly, NiF-NCs delivered a *Q*_s_ of 136.8 C g^−1^ at 1 A g^−1^. The results validated that the incorporation of rGO into NiF-NPs resulted in higher current, better specific capacity and improved conductivity. Further, NiF-NCs were used to fabricate a hybrid supercapacitor device, which showed a *Q*_s_ of 100.6 C g^−1^ at 0.5 A g^−1^. The determined *E*_s_ of device was 20.6 Wh kg^−1^ and the *P*_s_ was 835.9 W kg^−1^. Furthermore, the CE was determined as 99% and the capacitance retention was 40% even after 10 000 GCD cycles. The synergistic effect of NiF-NPs and rGO increased the electrical conductivity, improved the specific capacity and resulted in better charge transfer kinetics. Thus, the remarkable electrochemical performance validates that the green-synthesized NiF-NCs are potential candidates for energy storage applications. Table 1 provides the comparison of the material synthesized in the present work with similar reported materials.

**Table 1:** Comparison of the material synthesized in the present work with similar reported materials.

Material	Electrolyte	Specific capacitance (F g^−1^)	Ref.
NiFe_2_O_4_	1 M KOH	113.6	45
NiO@NiFe_2_O_4_	1 M KOH	248.4	45
MnCo_2_O_4_@NiO-0.3	2 M KOH	140	46
rGO-NiFe_2_O_4_	1 M Na_2_SO_4_	210.9	47
NiFe_2_O_4_	4 M KOH	23.02	48
NiFe_2_O_4_/AC (NFO/CI)	4 M KOH	67.71	48
NiFe_2_O_4_/AC (NFO/CII)	4 M KOH	31.97	48
NiFe_2_O_4_/AC (NFO/CIII)	4 M KOH	36.49	48
rGO-NiFe_2_O_4_	1 M KOH	215.7	49
NiFe_2_O_4_	1 M Na_2_SO_4_	50	47
NiFe_2_O_4_ (NiF-NPs)	1 M KOH	88.38	This work
NiFe_2_O_4_@rGO (NiF-NCs)	1 M KOH	288.17	This work

## Conflicts of interest

The authors declare that they have no competing interests.

## Abbreviations

NiF-NPs: Nickel ferrite nanoparticles
NiF-NCs: Nickel ferrite with reduced graphene nanocomposites
CV: Cyclic voltammetry
GCD: Galvanostatic charge–discharge
EIS: Electrochemical impedance spectroscopy
HSC: Hybrid supercapacitor

## Data Availability

Data will be made available on request.
